# Dynamics of Systemic Inflammation as a Function of Developmental Stage in Pediatric Acute Liver Failure

**DOI:** 10.3389/fimmu.2020.610861

**Published:** 2021-01-15

**Authors:** Yoram Vodovotz, Derek Barclay, Jinling Yin, Robert H. Squires, Ruben Zamora

**Affiliations:** ^1^ Department of Surgery, University of Pittsburgh, Pittsburgh, PA, United States; ^2^ Center for Inflammation and Regeneration Modeling, McGowan Institute for Regenerative Medicine, Pittsburgh, PA, United States; ^3^ Pittsburgh Liver Research Center, University of Pittsburgh, Pittsburgh, PA, United States; ^4^ Department of Pediatrics, University of Pittsburgh, Pittsburgh, PA, United States

**Keywords:** systems biology, inflammation, serum, biomarker, network analysis

## Abstract

The Pediatric Acute Liver Failure (PALF) study is a multicenter, observational cohort study of infants and children diagnosed with this complex clinical syndrome. Outcomes in PALF reflect interactions among the child’s clinical condition, response to supportive care, disease severity, potential for recovery, and, if needed, availability of a suitable organ for liver transplantation (LTx). Previously, we used computational analyses of immune/inflammatory mediators that identified three distinct dynamic network patterns of systemic inflammation in PALF associated with spontaneous survivors, non-survivors (NS), and LTx recipients. To date, there are no data exploring age-specific immune/inflammatory responses in PALF. Accordingly, we measured a number of clinical characteristics and PALF-associated systemic inflammatory mediators in daily serum samples collected over the first 7 days following enrollment from five distinct PALF cohorts (all spontaneous survivors without LTx): infants (INF, <1 year), toddlers (TOD, 1–2 years.), young children (YCH, 2–4 years), older children (OCH, 4–13 years) and adolescents (ADO, 13–18 years). Among those groups, we observed significant (P<0.05) differences in ALT, creatinine, Eotaxin, IFN-γ, IL-1RA, IL-1β, IL-2, sIL-2Rα, IL-4, IL-6, IL-12p40, IL-12p70, IL-13, IL-15, MCP-1, MIP-1α, MIP-1β, TNF-α, and $\text{N}{\text{O}}_{2}^{-}/\text{N}{\text{O}}_{3}^{-}$. Dynamic Bayesian Network inference identified a common network motif with HMGB1 as a central node in all sub-groups, with MIG/CXCL9 being a central node in all groups except INF. Dynamic Network Analysis (DyNA) inferred different dynamic patterns and overall dynamic inflammatory network complexity as follows: OCH>INF>TOD>ADO>YCH. Hypothesizing that systemically elevated but sparsely connected inflammatory mediators represent pathological inflammation, we calculated the *AuCon* score (area under the curve derived from multiple measures over time divided by DyNA connectivity) for each mediator, and identified HMGB1, MIG, IP-10/CXCl10, sIL-2Rα, and MCP-1/CCL2 as potential correlates of PALF pathophysiology, largely in agreement with the results of Partial Least Squares Discriminant Analysis. Since NS were in the INF age group, we compared NS to INF and found greater inflammatory coordination and dynamic network connectivity in NS vs. INF. HMGB1 was the sole central node in both INF and NS, though NS had more downstream nodes. Thus, multiple machine learning approaches were used to gain both basic and potentially translational insights into a complex inflammatory disease.

## Introduction

The Pediatric Acute Liver Failure (PALF) Study Group is the first and only multi-center, multi-national collaboration to examine PALF, a complex, devastating, rapidly evolving clinical syndrome whose onset is not completely understood (1, 2). Outcomes in PALF reflect a complex interaction among the child’s clinical condition, response to supportive care, disease severity, potential for recovery, and availability of a suitable organ if liver transplantation (LTx) is deemed life-saving (1, 2). Specific diagnostic and therapeutic targets and tools to predict death/spontaneous survival or to inform LT decisions in PALF are not available. In prior studies, we demonstrated that individuals with PALF exhibit variable systemic inflammatory responses. Traditional statistical analyses of these dynamic responses could not always distinguish spontaneous survivors (S) from non-survivors (NS) or LTx recipients; however, computational analyses of principal inflammatory drivers and dynamic networks did discriminate among these PALF sub-groups (3–5). These analyses also suggested the presence of overly robust, self-sustaining, and highly connected inflammation networks in NS. Furthermore, our study suggested a common inflammatory network regulated *via* HMGB1 and the chemokines IP-10 and MIG, which may represent a general “liver signature” in inflammatory conditions such as PALF, or which may represent the central contribution of the liver to other inflammatory diseases (4). Recently, we suggested that the inflammatory dynamic networks, and in particular HMGB1 connectivity, are dependent on (or reflect) the use of N-acetylcysteine (NAC) in the context of APAP toxicity in PALF patients (5). Furthermore, in isolated mouse hepatocytes (HC), high APAP dose led to a much more complex dynamic networks in C57BL/6 HC than in cells from HMGB1^-/-^ animals, suggesting that HMGB1 plays a central role in orchestrating the inflammatory response to APAP (5).

Animal models, human *in vitro* studies, and human observational studies have led to an increased understanding of the ontogeny of the complex immune response in humans that include age-dependent innate responses to damage-associated molecular patterns (DAMPs) and pathogen-associated molecular patterns (PAMPs), impaired Th17 and Th1 responses, and altered cytokine responses to infections (6). The increased frequency of infectious causes of PALF in infants compared to older children may reflect these differences. The role of an age-related immune/inflammatory response associated with non-infectious causes of PALF (e.g., metabolic, immune mediated, indeterminate) is unknown. We hypothesize that immune/inflammatory dysregulation occurs in PALF that is markedly dependent and/or reflective of developmental stage of immune response. We further hypothesize that no single inflammatory mediator determined at a single time-point along the dynamic PALF trajectory would be sufficient to characterize this complex syndrome and to predict outcome reliably. Rather, complex networks among various mediators over time are more likely to reflect dynamic changes in the systemic inflammatory response. A key, unresolved question concerns the impact of age, as a surrogate marker for immune development and response, on PALF outcomes and course of inflammation. Given the dynamic complexity of inflammation (7, 8), we thought to utilize a suite of machine learning approaches to delineate the dynamics, principal drivers, and interconnected networks of systemic inflammation in five PALF cohorts (all survivors who recovered spontaneously without liver transplant): infants (<1 year, INF), toddlers (1–2 years TOD), young children (2–4 years, YCH), older children (4–13 years, OCH) and adolescents (13–18 years, ADO).

## Materials and Methods

### Criteria for Patient Selection

**Figure 1:**
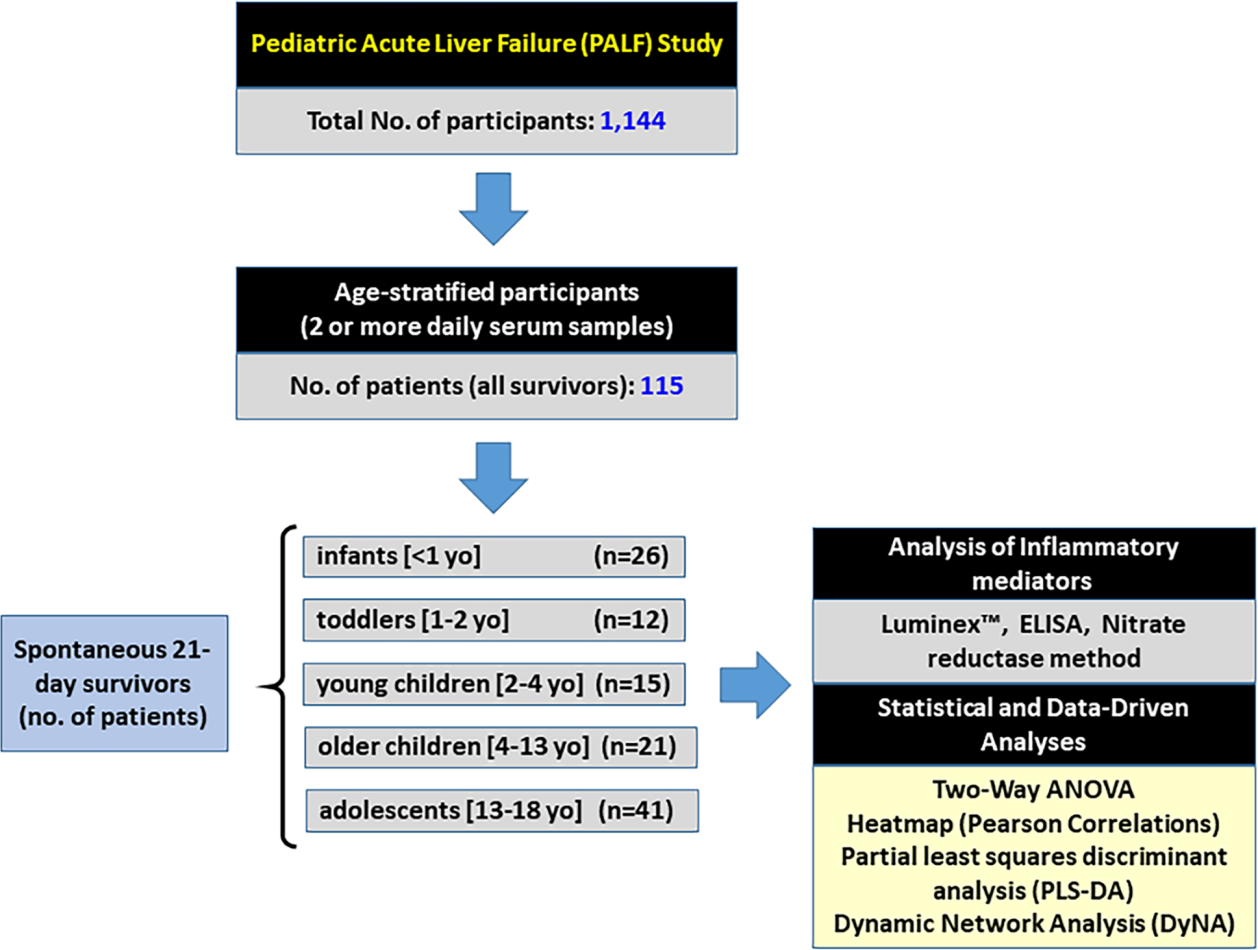
Flowchart of recruitment and PALF study participation. From a large cohort of 1,144 participants in the PALF study, 115 spontaneous survivors (determined at 21 days following enrollment without need for liver transplant) were selected if they had at least 2 or more daily samples with at least 100 µl of serum available. Patients were divided into five sub-groups based on age at the time of enrollment as indicated, and serum samples collected per protocol were assayed for a number of immune and inflammatory markers as described in *Materials and Methods*.

This work is part of a multi-center study conducted through the Pediatric Acute Liver Failure (PALF) Consortia (National Institutes of Health/National Institutes of Diabetes, Digestive, and Kidney Disease: 5U01 DK072146). The study was performed in accordance with all relevant guidelines and regulations and was approved by the Institutional Review Boards from all participating institutions (listed in the **Acknowledgments**), with written informed consent from parents and/or legal guardians and Certificate of Confidentiality provided by NIH. At the time of this analysis, PALF study enrollment included a total of 1,144 participants less than 18 years of age who met the following entry criteria: 1) no known evidence of chronic liver disease, 2) biochemical evidence of acute liver injury, and 3) hepatic-based coagulopathy (not corrected with vitamin K) defined as a prothrombin time (PT) ≥ 15 s or international normalized ratio (INR) ≥ 1.5 in the presence of clinical hepatic encephalopathy (HE), or a PT ≥ 20 s or INR ≥ 2.0 regardless of the presence or absence of HE (9, 10). In the present study, all patients who met the PALF entry criteria were alive with their native liver at 21 days after enrollment. Serum samples were collected on the calendar day of enrollment (d0) or with the first morning blood draw following enrollment and daily for up to seven days (d1-d7). Since not all patients had research samples obtained on all of the same days, patients were selected if they had at least two daily samples with at least 100 µL of serum available and fell into any of the age sub-groups as indicated in **Figure 1**. Serum samples were promptly frozen at −80°C at the enrollment site and later batch-shipped to the research biorepository for long-term storage before analysis.

Age classification is arbitrary and often dependent upon the underlying question(s) the Organization or research project needs to address. For example, a Position Paper from the World Health Organization to assess drug dosing administration classifies a child as anyone <19 years with subcategories of neonate (0–30 d), infant (1 mo to 2 years), young child (2–6 years), Child (6–12 years), and adolescent (12–18 years) (11). The Federal Drug Administration uses the following age categories for pediatric drug trials: Neonate (0 to 1 mo), Infants (1 mo to 2 years), Child (2–12 years), and Adolescent (12–16 years) (12). In guiding parents in understanding neurodevelopment and psychosocial progress for their children, the American Academy of Pediatrics use a classification of Baby (0–12 mo), Toddler (1–3 years), Pre-school (3–5 years), Grade-schooler (5–12 years), Teen (12–18 years) (11). Important, age-specific diagnoses in PALF were identified between children <90 days, 91 days–3 years and 4–17 years (13). We recognize that age is an unsatisfying surrogate for physiologic and immunologic ontogenesis as well as the range of xenobiotic and infectious exposures encountered between infancy and adolescence. We hypothesized that a granular assessment across the 3 age-specific PALF diagnostic categories, particularly within younger children, would more likely identify age-associated differences in the immune and inflammatory response in PALF. Age categories used for this analysis were classified as Infants (< 1 year), Toddler (1 to < 2 years), Young Children (2 to < 4 years), Older Children (4 to < 13 years), and Adolescents (13–18 years).

### Assays of Inflammatory Mediators

We measured a number of inflammatory mediators including cytokines and chemokines, HMGB1, and reactive nitrogen oxide species that serve as biomarkers for the complex inflammatory response using the Luminex™ 100 IS system (Luminex™, Austin, TX) and the Human 25-plex^®^ Luminex™ beadset (Millipore, Billerica, MA). The cytokines/chemokines included Eotaxin, GM-CSF, IFN-α2, IFN-γ, IL-1β, IL-1 receptor antagonist (IL-1RA), IL-2, soluble IL-2 receptor α chain (sIL-2rα), IL-4, IL-5, IL-6, IL-7, IL-8, IL-10, IL-12p40, IL-12p70, IL-13, IL-15, IL-17, IP-10, MCP-1, MIG, MIP-1α, MIP-1β, and TNF-α. HMGB1 was assayed by ELISA (Shino-Test, Kanagawa, Japan) and the NO reaction products $\text{N}{\text{O}}_{2}^{-}+\text{N}{\text{O}}_{3}^{-}$ were assayed using the nitrate reductase method (Cayman Chemical, Ann Arbor, MI).

### Statistical Analyses

All statistical analyses were carried out using SigmaPlot (Systat Software, San Jose, CA) as indicated. Due to non-normal data distribution, Kruskal-Wallis Analysis of Variance (ANOVA) on Ranks was used to compare clinical characteristics. Two-Way ANOVA followed by the Holm-Sidak *post hoc* test was used to analyze the time-dependent changes in inflammatory mediators across PALF sub-groups.

### Machine Learning Analyses

#### Variable Importance in Projection Scores in Partial Least Squares Discriminant Analysis

VIP scores measure the contribution that a variable makes to the PLS-DA model, a variant of PLS for classification purpose, which explains maximum separation between defined classes of samples. Thus, we used VIP scores to define variables that distinguish PALF patient sub-groups. The VIP score of a variable is calculated as a weighted sum of the squared correlations between the PLS-DA components and the original variable. The weights correspond to the percentage variation explained by the PLS-DA component in the model. The number of terms in the sum depends on the number of PLS-DA components found to be significant in distinguishing the classes. VIP scores indicate the importance of each variable in the projection used in a PLS-DA model and is often used for variable selection (14). The variables with the highest VIP scores are thus the most contributory variables in class discrimination. Many studies use VIP values of 1 or 2 for further data analysis, but this cut-off largely depends on the number of variables used (15). In the present study, the number of variables (inflammatory mediators) is relatively small, so we used a VIP cutoff of 1.0. To calculate the VIP scores for all variables (inflammatory mediators), we employed MetaboAnalyst (https://www.metaboanalyst.ca), a web-based tool suite developed for comprehensive metabolomic data analysis that also supports a wide array of functions for statistical, functional, as well as data visualization tasks (16, 17).

#### Dynamic Bayesian Network Inference

Network inference using inflammatory mediator data was carried out in MATLAB^®^ (The MathWorks, Inc., Natick, MA), using a Dynamic Bayesian Network (DBN) algorithm adapted from Grzegorczyk & Husmeier (18) and used by our group in previous studies (3–5, 19–21). Given time-series data, DBN analysis provides a way of inferring causal relationships among variables (e.g. inflammatory mediators) based on probabilistic measure. Unlike standard correlative approaches, DBNs consider the joint distribution of the entire dataset when making inferences about the dependencies between variables or nodes in the network. The values of each node are assumed to be distributed according to a chosen model (e.g. Gaussian) and the relationships among nodes are defined by the structure of the directed network and the corresponding conditional probability distributions of the interacting nodes. Network structure is inferred by a sampling technique that iteratively proposes candidate structures and evaluates them based on how well they fit the observed data using a specified scoring criterion, until reaching convergence on a network structure with the highest score. The algorithm uses an inhomogeneous dynamic changepoint model, with a Bayesian Gaussian with score equivalence (BGe) scoring criterion. The output of the aforementioned algorithm is a final graph structure indicating the interactions. In this analysis, time courses of unprocessed inflammatory mediator measurements from each patient were used as input for the DBN inference algorithm as described.

#### Dynamic Network Analysis

DyNA was carried out to define, in a granular fashion, the central inflammatory network nodes as a function of both time and PALF patient sub-group. Since DyNA also allows for granular temporal resolution of networks over distinct time intervals, using inflammatory mediator measurements for each patient, networks were created over seven consecutive time periods (d0–d1, d1–2, d2–3, d3–4, d4–5, d5–6, d6–7) using MATLAB^®^ software as described previously (3, 4, 22). Connections, defined as the number of trajectories of serum inflammatory mediators that move in parallel (black edges) or in anti-parallel (red edges) fashion across time intervals, were created if the Pearson correlation coefficient between any two nodes (inflammatory mediators) at the same time-interval was greater or equal to a threshold ranging from an absolute value of 0.7 (a correlation value commonly used to characterize trajectories that move in parallel either up or down) to 0.95, as appropriate. The network complexity for each time-interval was calculated using the following formula: Sum (N_1_ + N_2_ +…+ Nn)/(n − 1), where N represents the number of connections for each mediator and n is the total number of mediators analyzed.

#### Area Under the Curve and Connectivity Score

The **AuCon** score was developed to test the hypothesis that mediators that are present at high systemic levels but with low interconnectivity might represent pathological inflammatory processes, and thus may serve as possible disease biomarkers. In contrast, mediators present at low levels in the circulation and that are highly connected were hypothesized to be part of an adaptive and/or beneficial response to liver injury. Accordingly, **AuCon** segregates mediators based on their circulating levels over time (as defined by the area under the curve [AUC]), corrected for their dynamic connectivity (N, defined by DyNA as described above). The **AuCon** score for each inflammatory mediator (i) was calculated as follows: **AuCon_i_ = log_10_ [(AUC_i_/(1+N_i_)]**.

## Results

### Clinical Outcomes Differ as a Function of Age in Pediatric Acute Liver Failure Patients

From 1,144 PALF study participants, we identified 115 spontaneous survivors that met the inclusion and exclusion criteria described above. The participants were then separated by age-group in five distinct PALF cohorts as shown in **Figure 1**. Age distribution and clinical characteristics at enrollment for PALF Study participants used in this study are shown in **Table 1**. Interestingly, of the six clinical characteristics compared (ALT, INR, total bilirubin, creatinine, venous ammonia, and encephalopathy) there were statistically significant differences in ALT and creatinine among all age-groups (**Table 1**).

**Table 1:** Age distribution and clinical characteristics at enrollment for PALF Study participants.

Patient sub-group	No. of patients	Sex (M/F)	Age, mean (yr)	Age, median (yr)	Q1–Q3
infants (**INF**, <1 year)	26	19/7	0.13	0.04	0.03–0.12
toddlers (**TOD**, 1–<2 years) young children (**YCH**, 2–<4 years)	12 15	8/4 10/5	1.39 3.17	1.39 3.13	1.20–1.54 2.76–3.73
older children (**OCH**, 4–<13 years)	21	11/10	7.47	6.68	5.43–9.75
adolescents (**ADO**, 13−18 years)	41	14/27	15.78	15.84	14.6–17.4
**Patient sub-group**	**INF** <1 year	**TOD** 1–<2 years	**YCH** 2–<4 years	**OCH** 4–<13 years	**ADO** 13–18 years
*ALT (IU/L) **INR** **Total bilirubin (mg/dL)** *Creatinine (mg/dL) **Venous ammonia (µmol/L)** **Hepatic encephalopathy (n)** **Grade 0** **Grade I** **Grade II** **Grade III** **Grade IV** **Unknown** **Not assessable**	**1134.5 ± 464** 3.12 ± 0.2 8.65 ± 1.4 **0.39 ± 0.05** 55.56 ± 7.7 11 (42.3%) 4 (15.4%) 0 (0.0%) 0 (0.0%) 0 (0.0%) 5 (19.2%) 6 (23.1%)	**3912.3 ± 929** 3.30 ± 0.51 6.33 ± 1.3 **0.39 ± 0.07** 92.67 ± 21.03 4 (33.3%) 1 (8.3%) 2 (16.7%) 1 (8.3%) 0 (0.0%) 2 (16.7%) 2 (16.7%)	**4401.7 ± 1061** 3.19 ± 0.54 7.03 ± 1.83 **0.59 ± 0.22** 52.54 ± 9.11 6 (40.0%) 3 (20.0%) 2 (13.3%) 0 (0.−0%) 0 (0.0%) 2 (13.3%) 2 (13.3%)	**4006 ± 650** 2.40 ± 0.23 7.71 ± 1.66 **0.62 ± 0.13** 44.08 ± 6.09 10 (47.6%) 5 (23.8%) 1 (4.8%) 1 (4.8%) 1 (4.8%) 2 (9.5%) 1 (4.8%)	**4765 ± 614** 3.04 ± 0.23 5.17 ± 0.93 **0.87 ± 0.08** 71.74 ± 7.8 28 (68.3%) 5 (12.2%) 2 (4.9%) 1 (2.4%) 0 (0.0%) 5 (12.2%) 0 (0.0%)
*significant difference among groups by Kruskal-Wallis Analysis of Variance (ANOVA) on Ranks					
**Patient sub-group**	**INF** <1 year	**TOD** 1–<2 years	**YCH** 2 to <4 years	**OCH** 4–<13 years	**ADO** 13–18 years
**Diagnoses, n (%)** **Indeterminate** **Acetaminophen (APAP)** **APAP**—**acute toxicity** **APAP**—**chronic exposure** **APAP**—**therapeutic misadventure** **Adenovirus** **Enterovirus/Cocksackie/echovirus** **Autoimmune hepatitis** **Autoimmune marker positive** **Fructosemia** **Galactosemia** **Hemophagocytic lymphohistiocytosis (HLH)** **Ischemic hepatopathy/Sepsis/Cardiac** **Ischemia/shock** **Mitochondrial** **Neonatal hemochromatosis (NH)/GALD** **Herpes simplex** **Hepatitis E** **Drug-induced hepatitis** **Chronic hepatitis** **Urea cycle disorder** **Wilson**’**s disease** **Multiple** **Other diagnosis**	4 (15.4%) 1 (3.8%) – – – 1 (3.8%) 6 (23.1%) – – 1 (3.8%) 1 (3.8%) – 1 (3.8%) – – 3 (11.5%) 4 (15.4%) – – – – – – 4 (15.4%)	7 (5.8%) – – – – – – – 2 (16.7%) – – – 1 (8.3%) – – – – – – – – – – 2 (16.7%)	8 (53.3%) – – – 2 (13.3%) – – – 1 (6.7%) – – – 1 (6.7%) – 1 (6.7%) – – – 1 (6.7%) – – – – 1 (6.7%)	7 (33.3%) 1 (4.8%) – – 1 (4.8%) 1 (4.8%) – 2 (9.5%) 2 (9.5%) – – 1 (4.8%) – 1 (4.8%) – – – – 1 (4.8%) – – – 1 (4.8%) 3 (14.3%)	5 (12.2%) 10 (24.4%) 10 (24.4%) 1 (2.4%) – – – 2 (4.9%) 1 (2.4%) – – 2 (4.9%) 1 (2.4%) – – – – 1 (2.4%) 2 (4.9%) 1 (2.4%) 1 (2.4%) 1 (2.4%) 1 (2.4%) 2 (4.9%)

### Differential Dynamics of Systemic Inflammatory Mediators in Pediatric Acute Liver Failure Patients as a Function of Age

To assess the response and time-dependent changes in inflammatory mediators across the different PALF sub-groups (INF vs. TOD vs. YCH vs. OCH vs. ADO), we assayed a number of mediators that represent most of the major inflammatory and immune pathways. Multiple comparison of the time-courses in the five patient sub-groups by Two-Way ANOVA suggested significant changes in 17 inflammatory mediators (**Eotaxin, IFN-γ, IL-1RA, IL-1β, IL-2, sIL-2Rα, IL-4, IL-6, IL-12p40, IL-12p70, IL-13, IL-15, MCP-1, MIP-1α, MIP-1β, TNF-α,** and $NO_{2}^{-}/NO_{3}$) out of 27 over time. The time-courses of the inflammatory mediators for each patient sub-group and P values are shown in **Supplementary Figure 1**.

### Dynamic Bayesian Network Inference Identifies Both Common and Age-Distinct Nodes of Systemic Inflammation

**Figure 2:**
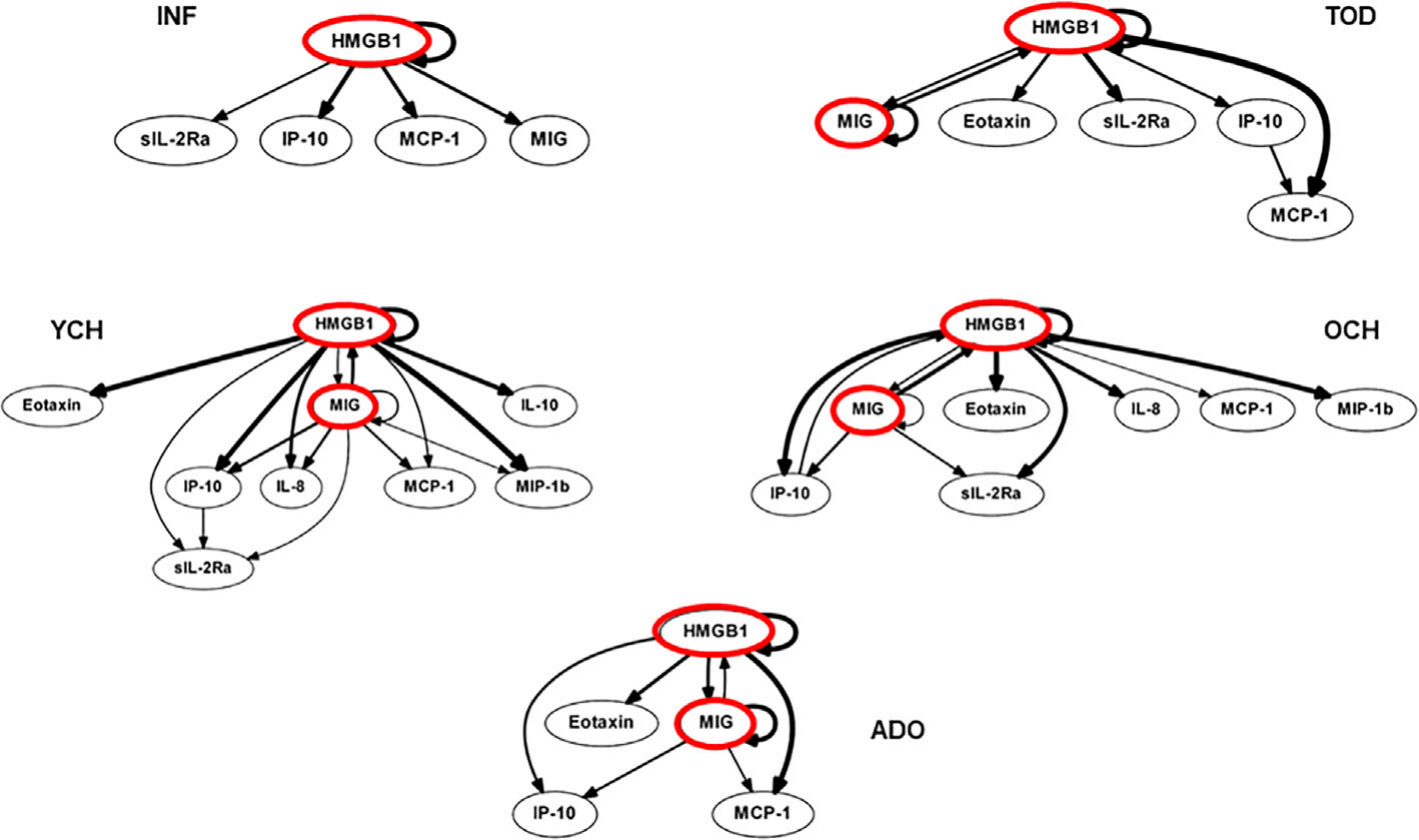
Dynamic Bayesian Network (DBN) analysis of circulating inflammatory mediators in PALF patients. Serum samples from PALF survivors were assessed for a number of inflammatory mediators and segregated into five sub‐groups (INF, TOD, YCH, OCH, and ADO) based on age as described in *Materials and Methods*. Inflammatory mediators are shown as nodes, and the arrows connecting them suggest an influence of one mediator on the one(s) to which it is connected. The arrows do not distinguish positive from negative influences of one mediator on another. Semi-circular arrows suggest either positive or negative feedback of a given mediator (highlighted in red) on itself.

We utilized DBN inference to determine if mediator feedback structures in inflammatory networks in PALF are related to or dependent on patient age. Similar to our previous studies in PALF, trauma, and sepsis (3, 4, 20, 22, 23), we focused on mediators that exhibit self-feedback as central nodes in the five age-subgroups of PALF survivors (INF, TOD, YCH, OCH, and ADO). Though data were segregated by age-group before being subjected to DBN inference, the algorithm did not make assumptions about the connectivity of the network in any sub-group. DBN inference suggested a primary network driven by two core motifs: **HMGB1** (in all subgroups) and **MIG (**in all sub-groups except INF, where HMGB1 was inferred to affect its downstream production). In addition, HMGB1 was inferred to affect the downstream production of **MCP-1** (in all sub-groups) and **sIL-2Rα (**in all sub-groups except ADO) (**Figure 2**).

### Variable Importance in Projection Scores in Partial Least Square Discriminant Analysis Identify Inflammatory Mediators That Serve as Major Discriminants in Age-Based Pediatric Acute Liver Failure Subgroups

**Figure 3:**
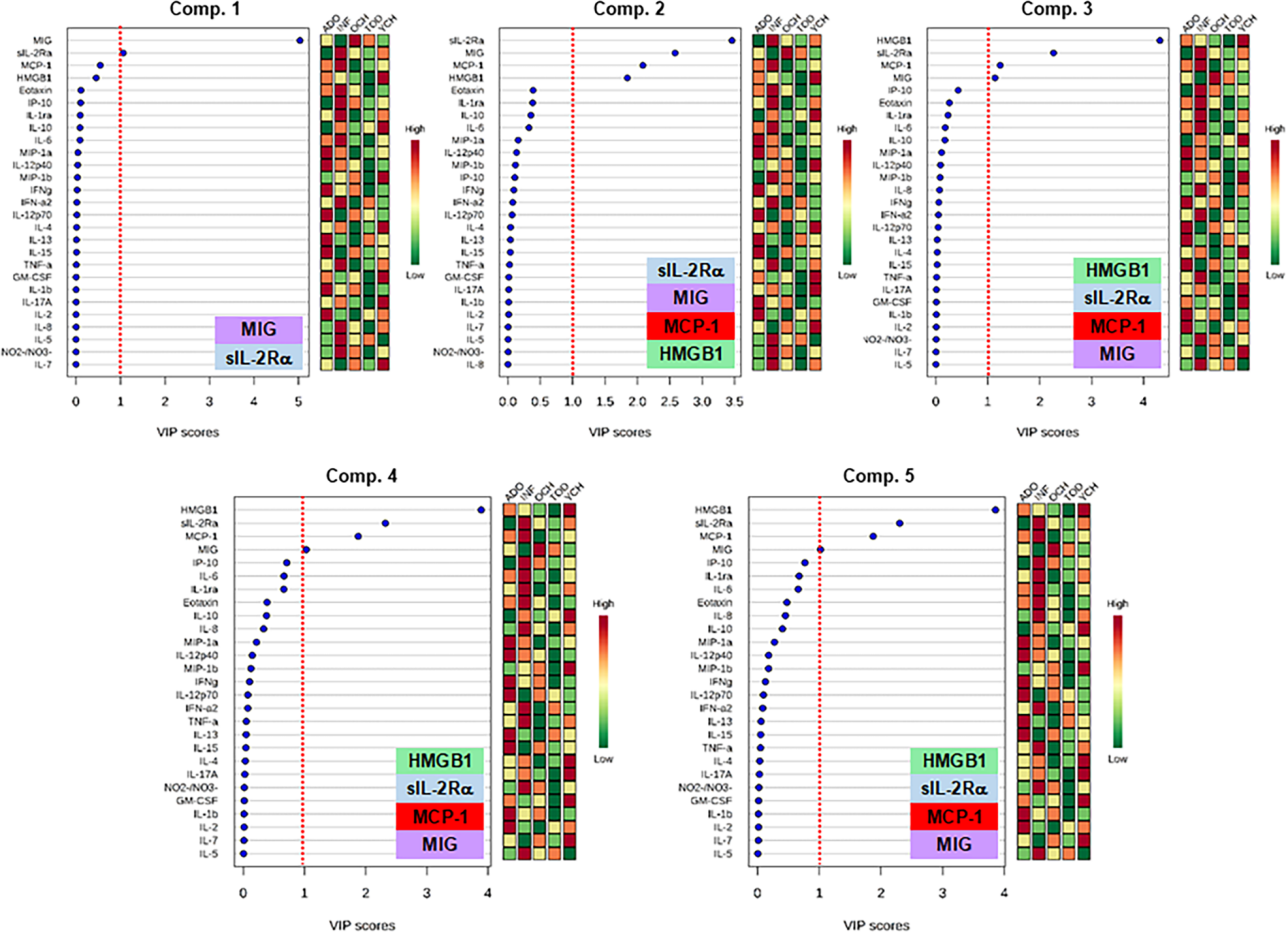
Variable Importance in Projection (VIP) scores in the Partial Least Square Discriminant Analysis (PLS-DA) of inflammatory mediators in PALF. Serum samples of five PALF patient sub-groups (INF, TOD, YCH, OCH, and ADO) were assayed for 27 inflammatory mediators using Luminex^®^ analysis as described in *Materials and Methods*. In order to estimate quantitatively the discriminatory power of each individual mediator, VIP scores in PLS-DA were calculated in the main 5 components using the web-based tool MetaboAnalyst. The graphs rank the inflammatory mediators according to their VIP scores (left) and the colored boxes in the right indicate the relative concentration of the corresponding mediator in the different patient sub-groups (INF, TOD, YCH, OCH, and ADO) as indicated. The insets show the mediators with the highest VIP scores (cutoff value: ≥ 1, red dotted line).

We next thought to examine quantitatively the discriminatory power of individual mediators by comparing the Variable Importance in Projection (VIP) scores in Partial Least Square Discriminant Analysis (PLS-DA). Ranking of the different mediators according to their VIP scores is shown in **Figure 3**. As a result of this analysis, the factors with the highest VIP scores (cutoff of 1.0) and thus the most contributory variables in class discrimination (INF vs. TOD vs. YCH vs. OCH vs. ADO) in the PLS-DA model were **MIG, sIL-2Rα, MCP-1**, and **HMGB1** (**Figure 3**).

### Dynamic Network Analysis Shows Differential Trajectories of Systemic Inflammation That Differentiate Pediatric Acute Liver Failure Age-Based Subgroups

**Figure 4:**
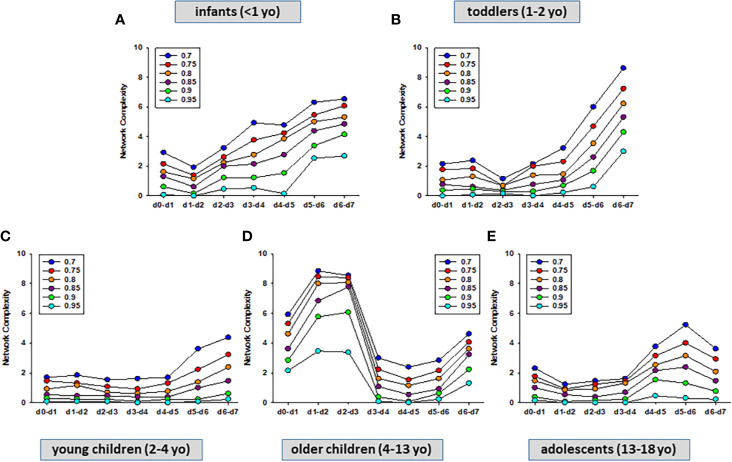
Dynamic Network Analysis (DyNA) of inflammatory mediators in PALF patients. Circulating inflammatory mediators in serum samples from PALF spontaneous survivors were segregated into five sub-groups (INF, TOD, YCH, OCH, and ADO) and DyNA (stringency level 0.7−0.95) was performed during each of the following seven time frames: d0−d1, d1−2, d2−3, d3−4, d4−5, d5−6, d6−7 as described in *Materials and Methods*. Panels **(A–E)** show the network complexity in INF **(A)**, TOD **(B)**, YCH **(C)**, OCH **(D)**, and ADO **(E)** calculated as described in *Materials and Methods*.

**Figure 5:**
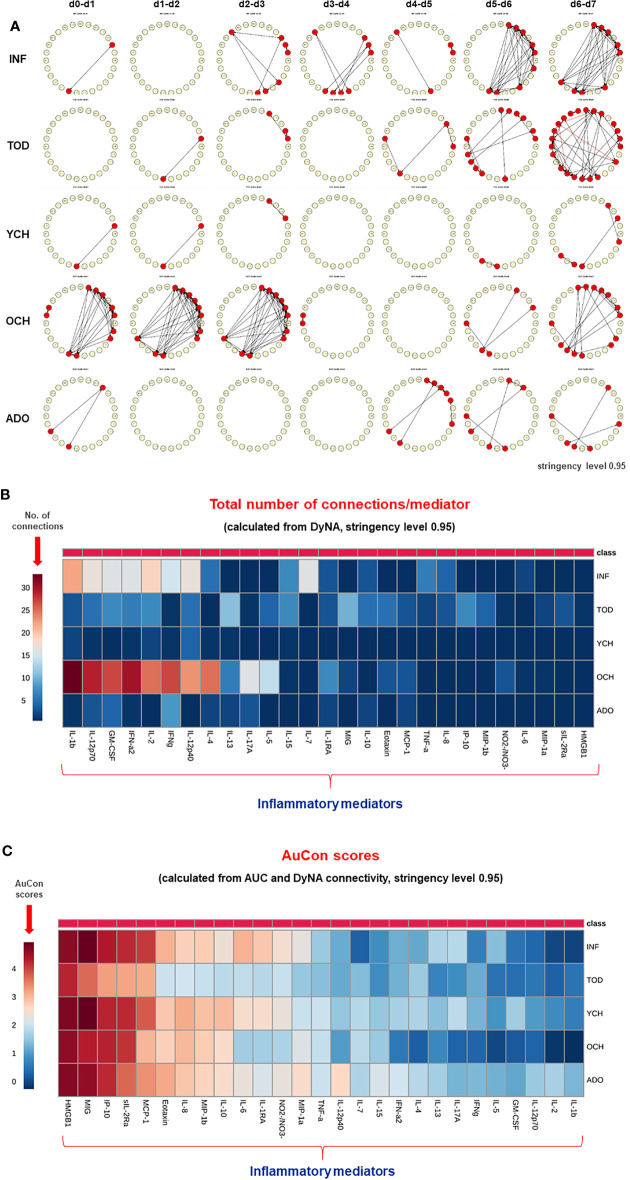
Differential Dynamic Network Connectivity in PALF patients. **(A)** An overview of all the dynamic networks (stringency level 0.95) and mediator connections over all time-intervals in five PALF patient sub‐groups (INF, TOD, YCH, OCH, and ADO) determined by DyNA as described in *Materials and Methods* and **Figure 4**. Closed red circles represent mediators with at least one connection to another mediator, while open yellow circles represent mediators that had no connections to other mediators. **(B)** Heatmap showing the total number of connections for each circulating inflammatory mediator determined by DyNA (stringency level 0.95) in five PALF patient sub‐groups (INF, TOD, YCH, OCH, and ADO) as described in *Materials and Methods*. **(C)** Heatmap showing the **AuCon** scores for each inflammatory mediator in five PALF patient sub‐groups (INF, TOD, YCH, OCH, and ADO) calculated from AUCs (**Supplementary Figure 2**) and DyNA connectivity (stringency level 0.95, **B**).

Previously, we showed that PALF patient sub-groups with different outcomes (4) or same outcome but different treatments (5) had different dynamic networks of inflammation as inferred using Dynamic Network Analysis (DyNA), an algorithm aimed at defining granular network connections over discrete time intervals (4, 5, 24). Accordingly, we hypothesized that employing the same methodology would also differentiate among the interconnections among inflammatory mediators in the five age-subgroups of PALF survivors. (INF, TOD, YCH, OCH, and ADO) over seven time frames (d0-d7) as described in *Materials and Methods*. In support of our hypothesis, DyNA suggested different dynamic patterns and overall dynamic inflammatory network complexity in all PALF sub-groups (**Figure 4**). This analysis, in which DyNA was performed at stringencies ranging from 0.7 to 0.95, suggested that dynamic network signatures were both robust and distinct across age groups. Accordingly, we focused on DyNA at a stringency level of 0.95 (**Figure 5A**). This analysis showed an overall different network patterns as well as marked differences in inflammatory mediators and overall network connectivity as a function of age, as follows: OCH (138) > INF (84) > TOD (53) > ADO (15) >YCH (numbers in parentheses indicate the total number of connections/patient subgroup. We also depict these results as a heatmap in **Figure 5B**. Based on this analysis, **IL-1β, IL-12p70, GM-CSF, IFN-α2**, **IL-2**, and **IFN-γ** were the mediators with the highest number of connections (**Figure 5B**), suggesting that inflammatory pathways associated with these mediators play significant roles in PALF survivors. Of note, those mediators were neither among the central nodes in the dynamic Bayesian networks (see **Figure 2** above) nor were among the mediators with VIP scores greater than 1 (see **Figure 3** above), possibly indicating secondary or parallel/orthogonal pathways operant in PALF patients who survive with their native liver to 21 days.

### *AuCon* Scores Serve to Differentiate Inflammatory Mediators Relevant in Pediatric Acute Liver Failure

**Figure 6:**
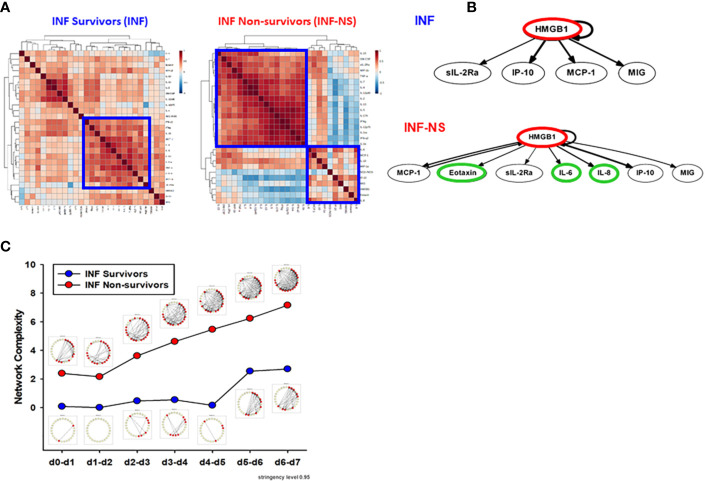
PALF non-survivors have greater inflammatory coordination and dynamic network connectivity than survivors in INF age-group. Serum samples from PALF survivors and non-survivors (all INF, <1 year) were assessed for 27 inflammatory mediators using Luminex^®^ analysis as described in *Materials and Methods*. **(A)** Spearman cross-correlation analysis of inflammatory mediators in INF (left panel) and INF-NS (right panel). **(B)** Dynamic Bayesian Network (DBN). Inflammatory mediators are shown as nodes, and the arrows connecting them suggest an influence of one mediator on the one(s) to which it is connected. The arrows do not distinguish positive from negative influences of one mediator on another. Semi-circular arrows suggest either positive or negative feedback of a given mediator (highlighted in red) on itself. **(C)** Network complexity of circulating inflammatory mediators in PALF patients (INF vs. INF-NS) determined by DyNA (stringency level 0.95).

We next hypothesized that mediators that are both present at higher levels in the serum over time, as well as being relatively less connected in dynamic networks, reflect pathology in PALF. Accordingly, we defined a novel metric based on the AUCs from the time courses (**Supplementary Figure 2**) along with the total number of connections (N) from DyNA (**Figures 5A, B** and **Supplementary Figure 2**) for each inflammatory mediator (i). This metric thus combines two independent analyses: the AUC representing the magnitude of the dynamic production/release of the mediators, and the number of DyNA connections across all time points representing network connectivity. We termed this score “**AuCon**”. The detailed AuCon scores for each mediator in each patient group are shown in **Figure 6**. Based on this analysis, **HMGB1, MIG, IP-10, sIL-2Rα,** and **MCP-1** had the highest AuCon scores, indicating various combinations of relatively high circulating levels over time along with relatively low or very low network connectivity in all PALF sub-groups (**Figure 5C**). We note that AuCon results were largely in line with the results of PLS-DA/VIP analysis. Taken together, we interpret these results as suggesting these mediators as potential correlates of differential systemic inflammatory trajectories in PALF as a function of age.

### Dynamic Network Analyses Clearly Differentiate Trajectories of Systemic Inflammation in Pediatric Acute Liver Failure Survivors vs. Non-Survivors

While only 22.6% (26/115) of survivors were in the INF age range, in the NS the majority of patients (66,7%, 8/12) was < 1 year. We therefore sought to compare inflammatory networks of INF (survivors) vs. NS (time-courses of inflammatory mediators are shown in **Supplementary Figure 3**). A Spearman cross-correlation analysis suggested different inflammatory programs in INF vs. NS, with the latter exhibiting two fairly distinct modules (**Figure 6A**). In contrast, the INF group had a more homogenous distribution with only one main inflammatory module (**Figure 6A**). Based on DBN inference (**Figure 6B**), the two groups were remarkably similar, with **HMGB1** as the sole central node in both INF and NS and with shared downstream nodes (**sIL-2Rα, IP-10, MCP-1,** and **MIG**). However, the NS DBN included additional downstream nodes (**eotaxin, IL-6,** and **IL-8**). In agreement with our prior studies (4, 5), DyNA network connectivity was higher in NS as compared to INF (**Figure 6C**). DyNA networks in NS consisted of 22/27 highly connected mediators, as compared to less complex networks with lower number of connected mediators in INF (15/27) described above and shown in **Supplementary Figure 4**.

## Discussion

The clinical trajectory of PALF is dynamic and likely multi-factorial, and thus the precise onset of disease is rarely identified (1). Outcomes vary among children with seemingly similar etiology, disease severity, and treatment; thus, additional factors such as age, being a proxy for developmental and physiological features unique to that age, are likely involved to explain these variations. Immune dysregulation is becoming increasingly evident in PALF and may be a principal mechanism for driving organ failure regardless of diagnosis (4, 25, 26). Our preliminary results suggested an iterative experimental and computational framework for discovery of novel clinical biomarkers and potential therapeutic targets in PALF (3–5). Age-associated changes in principal drivers and networks of inflammation in PALF are largely unknown, and a key question that remained unanswered was the role of age in the dynamics of inflammatory networks in PALF. We thought to explore this question using Luminex data from participants in five distinct PALF cohorts (all spontaneous survivors without LTx) segregated by age: INF, TOD, YCH, OCH, and ADO. Our findings suggest a role for age-associated maturity of immune system and differential inflammatory networks in the response to liver injury in PALF.

Our previous work has recurrently pointed to four mediators (**HMGB1, MIG, IP-10,** and **MCP-1)** as possible biomarkers in PALF (4, 5). In line with those results, the DBN pattern in PALF age-subgroups suggested a network regulated *via* switching between **HMGB1** and the chemokine **MIG**, each of which drives its own expression. More importantly, comparison of the VIP scores in PLS-DA revealed that those mediators, in addition of being central nodes of the inflammatory response and together with **MCP-1,** and **sIL-2Rα**, can serve to differentiate the different PALF sub-groups as a function of age. This method has been previously used to investigate the age-related metabolic changes in healthy children from 6 months through 4 years of age (27).

The inflammatory processes associated with PALF are dynamic and their complexity is the result of many immune/inflammatory networks and mediator interactions that still need to be identified or characterized. We have previously shown that PALF patient sub-groups with different clinical outcomes (4) or same outcome but different etiologies and treatments (5) had different dynamic networks of inflammation. The results presented here clearly suggest that the differences in interconnections among inflammatory mediators and resulting network complexity in PALF are also a function of developmental stage. The differences in overall dynamic inflammatory network complexity (OCH > INF > TOD > ADO >YCH) seem to clearly reflect the age dependence. In contrast, we cannot explain the lack of linearity as a function of age. It is important to note that the age range in OCH (9 years) is much larger than in INF, TOD and YCH (<1−2 years) and almost double than in ADO (5 years); thus, future studies with a larger number of patients will be necessary to address this question.

There is abundant literature regarding the relationship between abnormal levels of inflammatory cytokines/chemokines and several disease states both in experimental and clinical settings. Those inflammatory mediators include some that were assessed in the present study: **HMGB1, MIG, IP-10, sIL-2Rα,** and **MCP-1**. The prototypical damage-associated molecular pattern (DAMP) molecule **HMGB1** has been shown to contribute to the pathogenesis of sepsis, traumatic shock, autoimmune diseases, cancer, as well as hepatic steatosis and fatty liver disease (28). Experimentally, an **HMGB1** neutralizing chimeric antibody has been shown to attenuate drug‐induced liver injury and postinjury inflammation in mice (29), and an increasing number of experimental disease models responding to therapy targeting **HMGB1** have been reported (30). Liver failure has also been associated with elevated levels of both **MIG and IP-10** mRNA (31, 32), and circulating levels of both chemokines have been implicated in chronic hepatitis C (33). Similarly, highly elevatated levels of **MCP-1** haven been reported in livers from patients with fulminant hepatic failure (34). More recently, our collaborators in the PALF Study Group have identified **sIL-2R** as one of the biomarkers linked to activation of CD8+ lymphocytes that predict clinical outcomes in PALF (35). Our present results not only confirm previous observations from our group and others, but highlight the centrality of those five inflammatory mediators and suggest that a coordinated response involving those mediators is critical in liver diseases such as PALF.

In order to efficiently discover novel inflammatory interactions that suggest possible PALF biomarkers, we created a novel metric (the AuCon score), which combines both systemic levels of a given mediator over time (AUC) as well as dynamic network connectivity (from DyNA). Based on prior studies in which we demonstrated that the cytokine IL-6 was elevated but not connected in mice undergoing experimental trauma/hemorrhage (36), we designed the AuCon metric to test the hypothesis that mediators that are elevated systemically but only sparsely connected might differentiate the age-related trajectories of systemic inflammation of PALF. Ranking the AuCon scores in the five PALF patient sub-groups clearly highlighted five mediators (**HMGB1, MIG, IP-10, sIL-2Rα,** and **MCP-1**) that met the criteria above. Interestingly, four of these mediators (**MIG, sIL-2Rα, MCP-1** and **HMGB1**) were identified by VIP scores in PLS-DA as the most major contributors to sub-group separation. Further study is needed in order to determine if AuCon scores simply serve to differentiate inflammatory trajectories or if this metric could highlight distinct biological functions.

Our modeling methods are limited by the imperfect current understanding of the etiology of PALF and, similar to our previous PALF studies (3–5), there are some unavoidable limitations to the present work. First, as the exact onset date for PALF cannot be determined, we are unable to determine the onset for the systemic inflammation in PALF patients. Under ideal circumstances, serial time course data would be obtained from the same PALF patients but unfortunately, this is not always possible. We could only analyze 8 days of samples, so changes in dynamic networks either before or after this time period remain unidentified and warrant further investigation. The same applies to samples from participants with short, mild events or severe, rapid progression to death or transplantation that were excluded from this analysis given the analytical requirement for at least two or more available blood samples. We also note the relatively small number of patients in some sub-groups as well as the differences in age range discussed above.

In conclusion, our findings support and extend previous observations regarding the use of computational modeling in PALF. These tools served to identify immune/inflammatory networks and mediator interactions that distinguish among outcome and age groups in PALF. Specifically, our results demonstrate that a combination of the measurement of relevant inflammatory mediators in serum samples from PALF participants followed by computational analysis (developed for biologically complex and dynamic conditions (37–41) and correlation of biomarkers with clinical outcomes as a function of age, a combined approach that has proven its value in other clinical settings, might be used not only to differentiate patient etiologies but may lead to novel therapeutic opportunities for PALF.

## Data Availability Statement

The original contributions presented in the study are included in the article/**Supplementary Material**; further inquiries can be directed to the corresponding author.

## Ethics Statement

The studies involving human participants were reviewed and approved by the Institutional Review Boards from all participating institutions (listed in the Acknowledgments). Written informed consent to participate in this study was provided by the participants’ legal guardian/next of kin.

## Author Contributions

YV RS, and RZ conceived the study, and wrote and edited manuscript. DB and JY performed the analyses. All authors contributed to the article and approved the submitted version.

## Funding

This project was supported by a Multi-Center Group to Study Acute Liver Failure in Children (NIH/NIDDK grant UO1 DK072146) and by the National Institutes of Health (NIH) through Grant No. UL1TR001857 (award to RZ from CTSI, University of Pittsburgh).

## Conflict of Interest

The authors declare that the research was conducted in the absence of any commercial or financial relationships that could be construed as a potential conflict of interest.
