# Supplementary material for: Dynamics of Systemic Inflammation as a Function of Developmental Stage in Pediatric Acute Liver Failure
Source: Front Immunol. 2021 Jan 15;11:610861. doi: 10.3389/fimmu.2020.610861 (PMC7844097; doi:10.3389/fimmu.2020.610861)
Supplement: Supplementary file 1 [file DataSheet_1.pdf]

Supplementary Material

Supplementary Figures

Suppl. Fig. 1

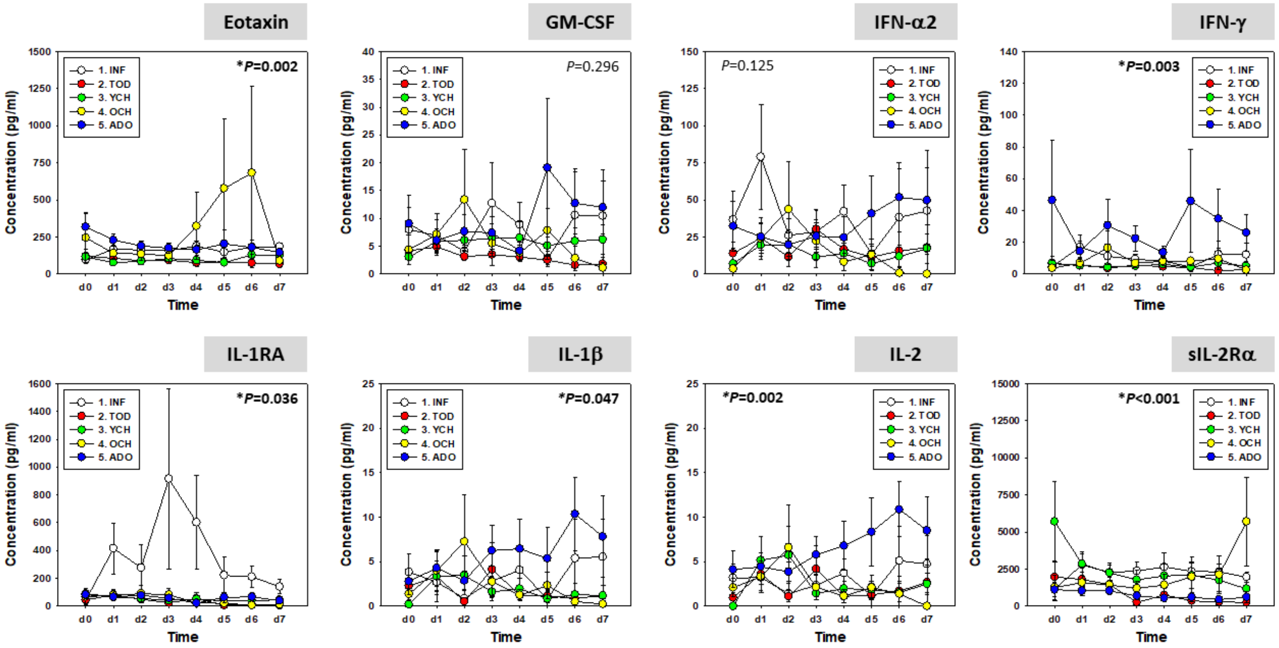

Suppl. Fig. 1

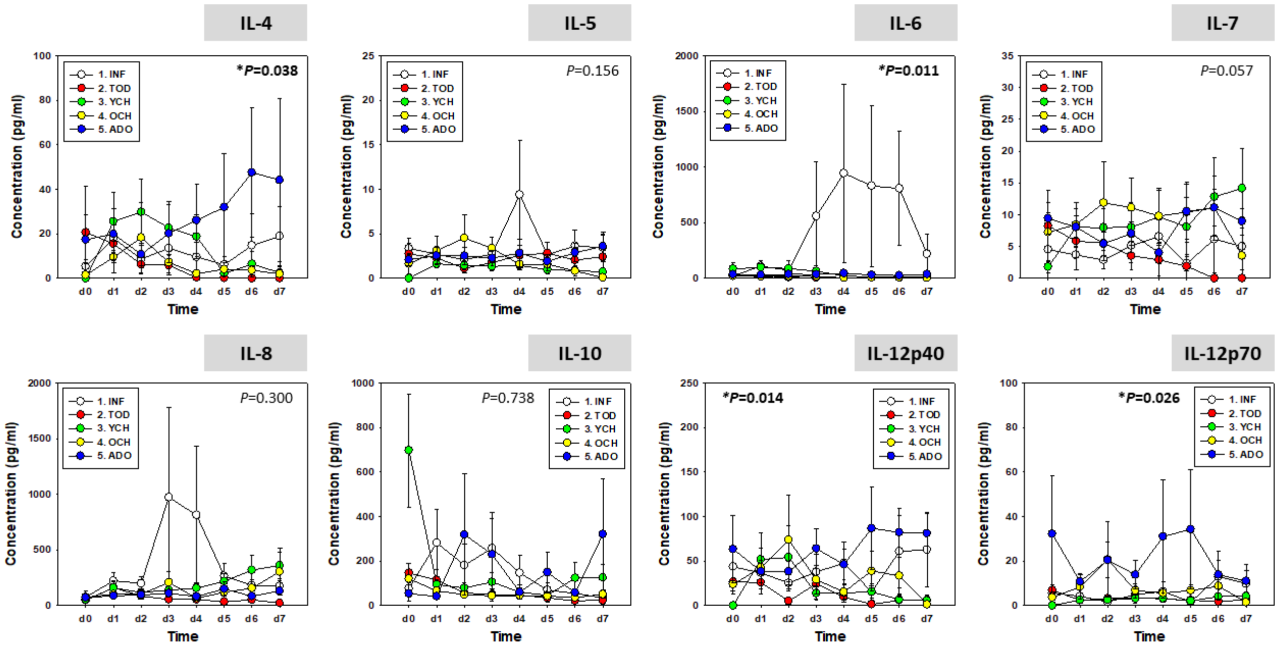

Suppl. Fig. 1

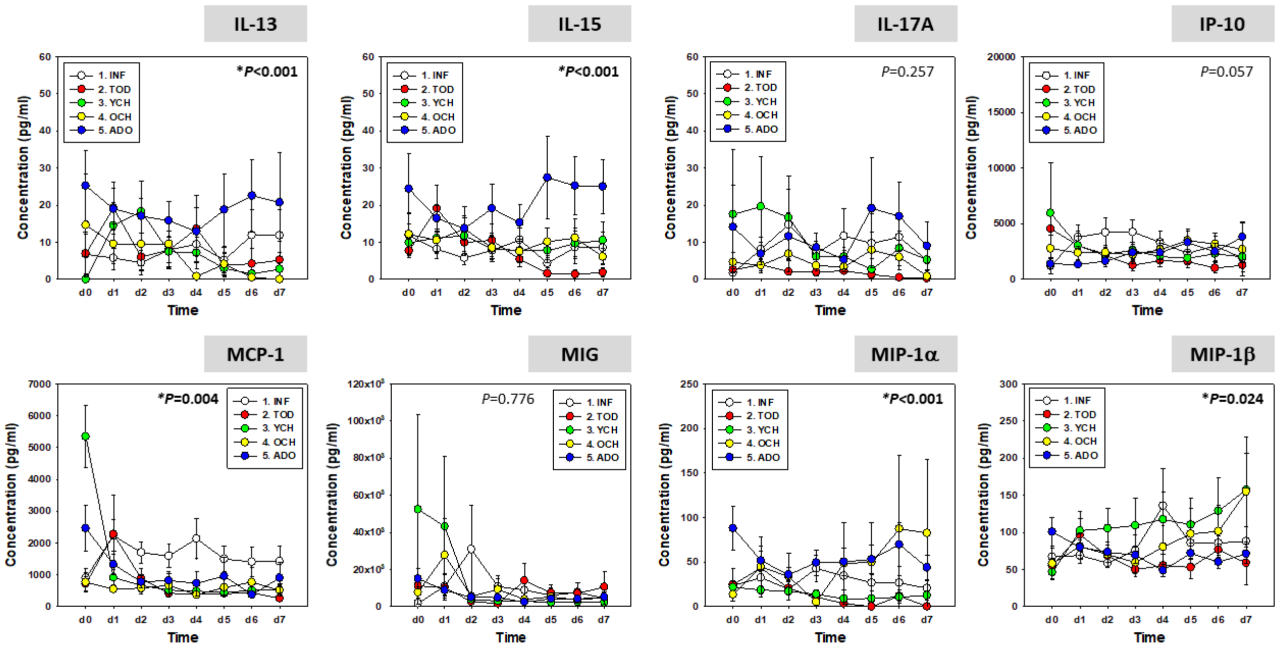

Suppl. Fig. 1

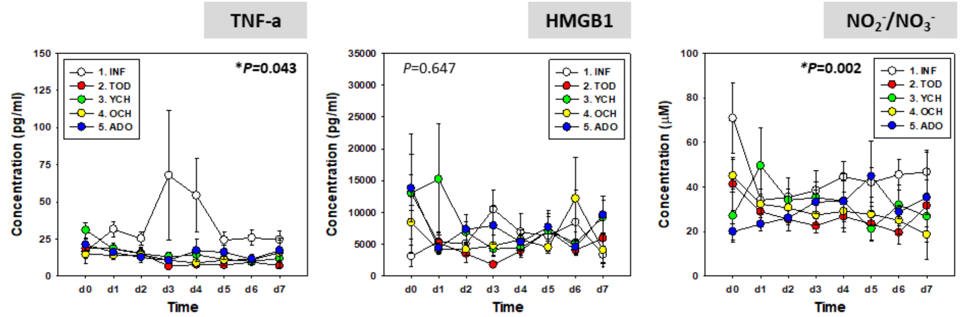

Supplementary Figure 1. Time-dependent release of inflammatory mediators in PALF patients. Serum samples from PALF survivors were assessed for a number of inflammatory mediators and segregated into five sub-groups (INF, TOD, YCH, OCH, and ADO) based on age as described in *Materials and Methods*. Results are presented as the mean ± SEM (significance level set at *P<0.05, analyzed by Two-Way ANOVA followed by Holm-Sidak post hoc test).

Suppl. Fig. 2

Mediator-AUC	INF	TOD	YCH	OCH	ADO	Total AUC
MIG	78235.88	53646.94	84664.61	61256.55	39300.04	317104.02
HMGB1	45889.44	35230.60	54306.82	41878.54	49000.44	226305.84
IP-10	22431.98	13571.98	18058.30	19040.11	16125.67	89228.05
sIL-2Ra	16276.65	5997.24	16160.31	13196.59	5176.81	56807.60
MCP-1	11702.17	5290.15	6453.53	4149.64	6656.05	34251.55
IL-8	2777.55	473.04	1312.32	942.83	705.00	6210.73
Eotaxin	1144.13	613.74	684.30	2147.70	1368.99	5958.86
IL-6	3439.11	78.21	335.27	81.78	235.19	4169.56
IL-1ra	2731.57	247.59	356.35	338.52	410.93	4084.97
IL-10	1046.53	406.68	919.55	367.37	1043.23	3783.36
MIP-1b	587.22	455.77	774.98	596.26	488.27	2902.50
IL-12p40	276.35	88.28	158.33	245.49	427.38	1195.82
NO2-/NO3-	298.89	183.13	232.66	203.94	217.51	1136.12
MIP-1a	204.40	102.21	94.78	316.49	375.43	1093.31
IFN-a2	266.89	125.49	95.24	113.53	228.15	829.30
TNF-a	249.53	78.00	103.28	84.80	103.05	618.66
IL-4	82.72	38.33	106.42	46.30	186.97	460.73
IFNg	70.64	30.59	38.14	58.87	197.65	395.89
IL-15	55.38	52.57	66.41	70.54	141.81	386.71
IL-13	53.92	60.73	53.05	41.18	128.88	337.76
IL-12p70	39.36	21.07	19.62	59.31	145.59	284.96
IL-17A	65.95	13.30	70.79	34.18	79.88	264.09
IL-7	31.47	23.81	62.52	68.06	55.35	241.22
GM-CSF	55.07	21.60	40.45	43.14	67.63	227.89
IL-2	20.91	14.73	18.89	17.67	46.35	118.57
IL-1b	21.28	12.94	13.17	18.79	40.82	107.00
IL-5	26.57	15.17	8.01	15.83	17.76	83.34

Total No. Connections/mediator	INF	TOD	YCH	OCH	ADO	Total No. Connections
IL-1β	22	3	2	33	1	61
IL-12p70	17	5	1	29	3	55
GM-CSF	16	7	0	27	4	54
IFN-α2	16	6	1	30	1	54
IL-2	19	7	2	25	0	53
IFNγ	15	1	1	27	8	52
IL-12p40	17	5	4	23	0	49
IL-4	5	0	1	25	2	33
IL-13	0	11	1	6	2	20
IL-17A	0	1	0	16	3	20
IL-5	0	4	0	14	0	18
IL-15	7	7	1	1	0	16
IL-7	16	0	0	0	0	16
IL-1RA	3	3	0	7	2	15
MIG	0	10	0	2	0	12
IL-10	3	5	0	0	2	10
Eotaxin	0	5	0	3	0	8
MCP-1	0	3	0	3	2	8
TNF-α	6	2	0	0	0	8
IL-8	4	3	0	0	0	7
IP-10	0	7	0	0	0	7
MIP-1β	0	4	0	0	0	4
NO_2-/NO_3-	0	1	0	3	0	4
IL-6	2	0	0	1	0	3
MIP-1α	0	2	0	1	0	3
sIL-2Rα	0	3	0	0	0	3
HMGB1	0	1	0	0	0	1

stringency level 0.95

Supplementary Figure 2. AUCs from the time courses of inflammatory mediators in five PALF subgroups (INF, TOD, YCH, OCH, and ADO) and total number of connections (N) from DyNA (stringency level 0.95) for each inflammatory mediator (i) as described in *Materials and Methods*.

Suppl. Fig. 3

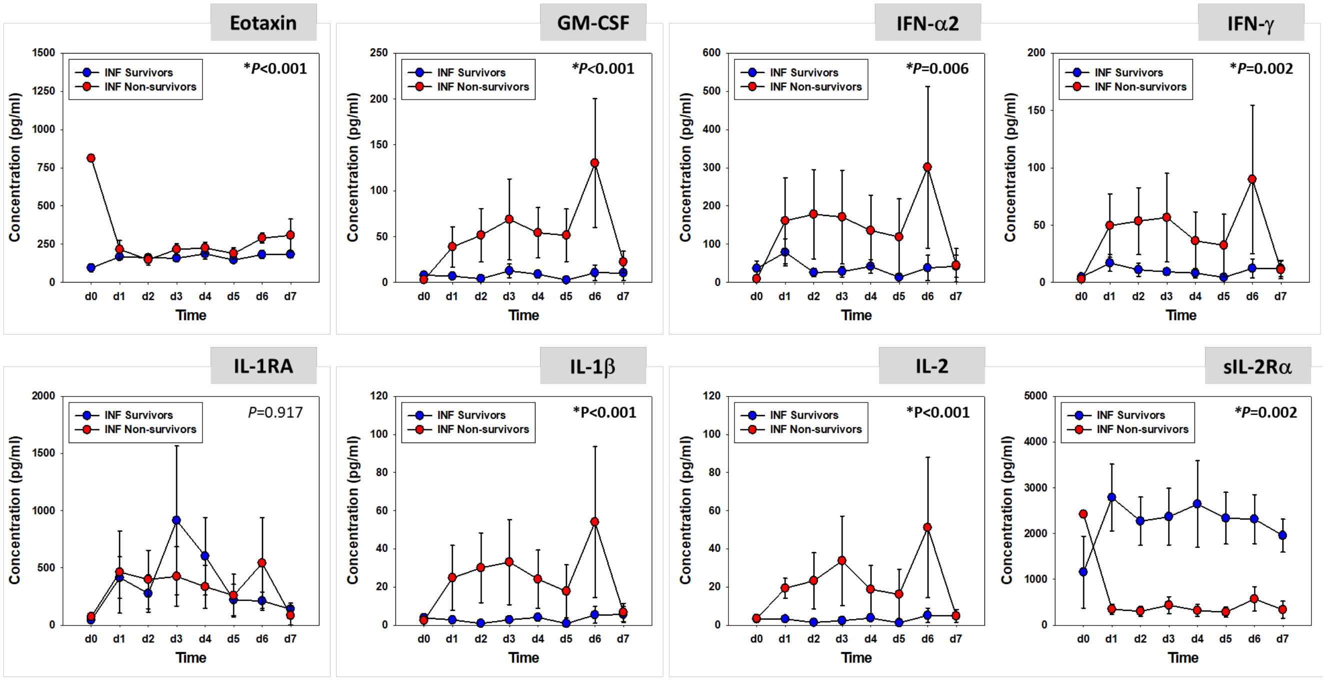

Suppl. Fig. 3

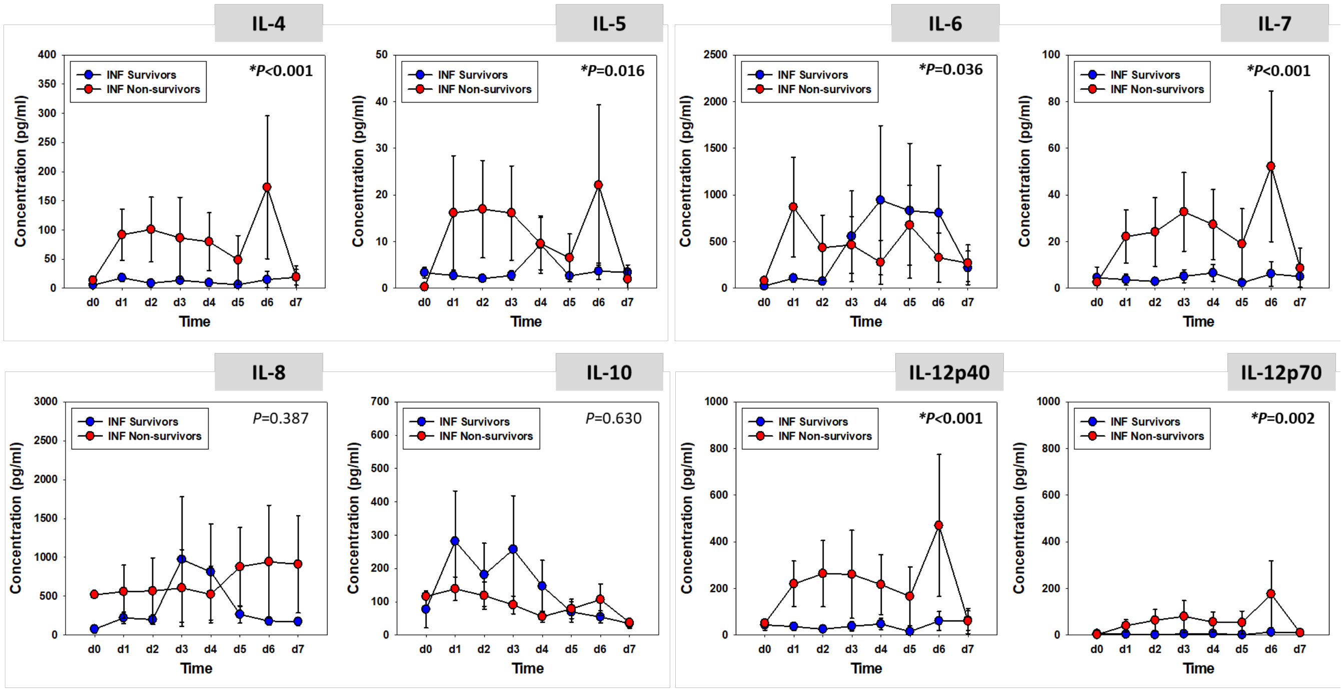

Suppl. Fig. 3

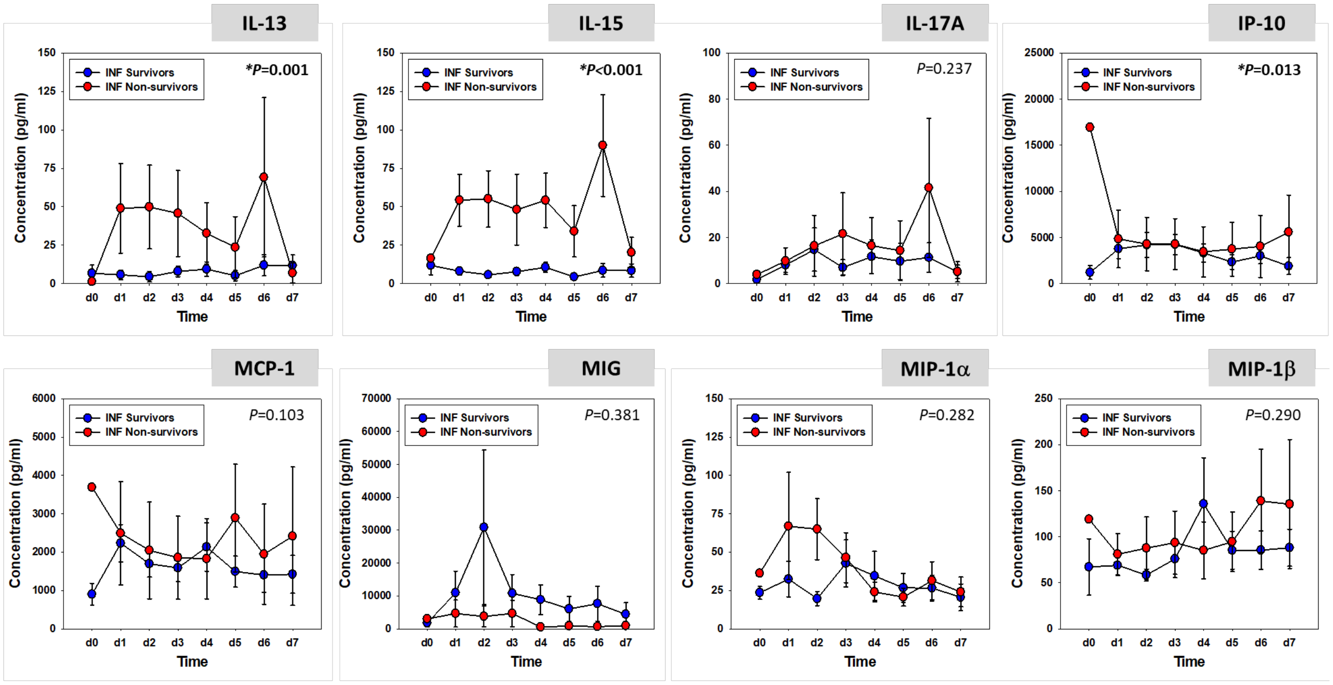

Suppl. Fig. 3

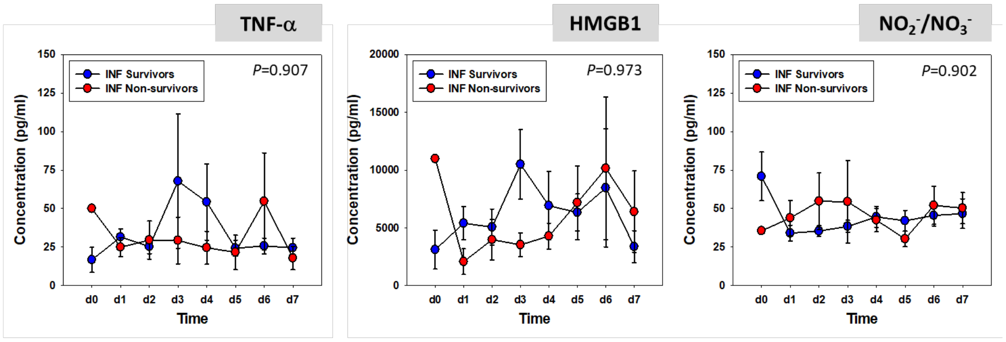

Supplementary Figure 3. Time-dependent release of inflammatory mediators in PALF patients. Serum samples from both INF survivors and non-survivors (NS) were assessed for a number of inflammatory mediators as described in *Materials and Methods*. Results are presented as the mean ± SEM (significance level set at *P<0.05, analyzed by Two-Way ANOVA followed by Holm-Sidak post hoc test).

Suppl. Fig. 4

INF	d0-d1	d1-d2	d2-d3	d3-d4	d4-d5	d5-d6	d6-d7	Total
IL-1b	1	0	1	3	1	8	8	22
IL-2	0	0	1	2	1	7	8	19
IL-12p40	0	0	0	2	0	7	8	17
IL-12p70	1	0	0	1	0	8	7	17
GM-CSF	0	0	0	0	0	8	8	16
IFN-a2	0	0	0	0	0	8	8	16
IL-7	0	0	0	0	0	8	8	16
IFNg	0	0	0	0	0	7	8	15
IL-15	0	0	0	0	0	0	7	7
TNF-a	0	0	3	2	1	0	0	6
IL-4	0	0	0	0	0	5	0	5
IL-8	0	0	2	1	1	0	0	4
IL-1ra	0	0	2	1	0	0	0	3
IL-10	0	0	1	2	0	0	0	3
IL-6	0	0	2	0	0	0	0	2
Eotaxin	0	0	0	0	0	0	0	0
sIL-2Ra	0	0	0	0	0	0	0	0
IL-5	0	0	0	0	0	0	0	0
IL-13	0	0	0	0	0	0	0	0
IL-17A	0	0	0	0	0	0	0	0
IP-10	0	0	0	0	0	0	0	0
MCP-1	0	0	0	0	0	0	0	0
MIG	0	0	0	0	0	0	0	0
MIP-1a	0	0	0	0	0	0	0	0
MIP-1b	0	0	0	0	0	0	0	0
HMGB1	0	0	0	0	0	0	0	0
NO2-/NO3-	0	0	0	0	0	0	0	0

NS	d0-d1	d1-d2	d2-d3	d3-d4	d4-d5	d5-d6	d6-d7	Total
IL-1b	8	6	8	11	12	12	13	70
IFN-a2	8	6	8	10	12	13	12	69
IL-13	6	5	6	11	12	12	13	65
IL-4	6	5	7	10	10	12	13	63
IL-12p40	3	4	9	10	12	12	13	63
IL-12p70	5	4	7	9	10	12	13	60
IFNg	4	2	7	9	10	12	13	57
IL-17A	0	2	7	10	11	13	13	56
IL-1ra	7	4	4	5	9	13	13	55
IL-7	2	3	6	8	12	12	12	55
IL-5	6	4	4	3	7	12	13	49
TNF-a	0	1	3	9	11	12	13	49
IL-2	0	1	3	5	12	12	13	46
GM-CSF	7	3	3	0	0	0	0	13
IL-8	0	2	3	3	1	0	2	11
IL-10	0	0	0	0	0	0	11	11
IP-10	0	2	3	3	0	0	3	11
IL-6	0	1	2	2	0	0	2	7
MCP-1	0	1	2	2	1	0	1	7
MIP-1b	0	0	0	0	0	3	0	3
MIG	0	0	1	0	0	0	0	1
NO2-/NO3-	0	0	1	0	0	0	0	1
Eotaxin	0	0	0	0	0	0	0	0
sIL-2Ra	0	0	0	0	0	0	0	0
IL-15	0	0	0	0	0	0	0	0
MIP-1a	0	0	0	0	0	0	0	0
HMGB1	0	0	0	0	0	0	0	0

stringency level 0.95

Supplementary Figure 4. Total number of connections (N) from DyNA (stringency level 0.95) for each inflammatory mediator in PALF (INF vs. INF-NS).
